# Pulsed Spray Pyrolysis for High-Quality Bismuth Ferrite Thin Films: Bi Content Enabled Tuning of Photoresponse, Ferroelectric Domains, and Charge Separation for Energy Harvesting Applications

**DOI:** 10.1021/acsomega.6c00654

**Published:** 2026-04-14

**Authors:** Nagashree Malur C, Haoze Zhang, Jan Seidel, Rajendra Bharathipura Venkataramana, Pankaj Sharma, Vinayak B. Kamble, Suresh D. Kulkarni

**Affiliations:** † Manipal Institute of Technology, 76793 Manipal Academy of Higher Education, Manipal 576104, India; ‡ College of Science and Engineering, 1065 Flinders University, Bedford Park, Adelaide, South Australia 5042, Australia; § ARC Centre of Excellence in Future Low-Energy Electronics Technologies, UNSW, Sydney, New South Wales 2052, Australia; ∥ School of Materials Science and Engineering, UNSW, Sydney, New South Wales 2052, Australia; ⊥ Flinders Institute for Nanoscale Science and Technology, 1065 Flinders University, Adelaide, South Australia 5042, Australia; # School of Physics, Indian Institute of Science Education and Research, Thiruvananthapuram 695551, India; ¶ Manipal Institute for Applied Physics, Manipal Academy of Higher Education, Manipal 576104, India

## Abstract

High-quality bismuth ferrite (BFO) thin films were prepared by using the pulsed spray pyrolysis technique with various bismuth-to-iron ratios. Our optimized pulsed deposition approach yielded dense, device-quality films suitable for large-scale applications. Morphological analysis confirmed the superior quality of the films produced. The bismuth content in the BFO significantly alters the ferroelectric polarization in the material as well as the photoresponse from the material. The Bi content variation results in the coexistence of Fe^3+^ and Fe^2+^ states, as confirmed by X-ray photoelectron spectroscopy (XPS), which has a significant impact on the material’s polarization. The 5 at % Bi-rich film exhibits larger ferroelectric domains compared to the stoichiometric BFO sample, with a transient responsivity of 0.173 mA/W. Furthermore, the fabrication of a single-layer device and the assessment of a suitable electrode for charge carrier separation were studied. The asymmetric electrode configuration enhanced the photoresponse of the device. The study highlights the potential of BFO to be incorporated into the perovskite solar cell device structure with Bi content playing a crucial role in controlling the functional properties of BFO.

## Graphical abstract

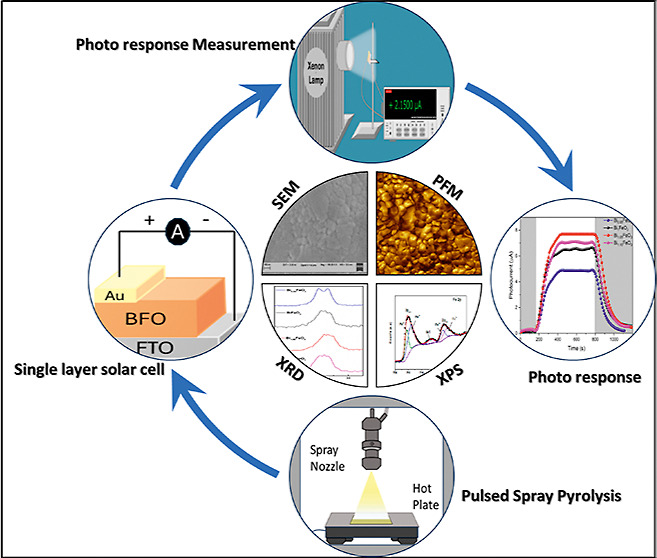

## Introduction

The advancement of sustainable and efficient photovoltaic materials has gained increasing importance in the pursuit of renewable energies. Among the promising candidates, ferroelectric oxides,^1^ particularly bismuth ferrite (BiFeO_3_, BFO), have emerged as materials of interest. Under ambient conditions, BFO’s structural stability and humidity resistance make it superior to conventional materials. Due to the ferroelectric polarization, the inbuilt potential helps in the separation of the charge carrier and results in above-bandgap open-circuit voltage^2^ and switchable photocurrent.^3^ As a result, BFO has become a prominent material for ferroelectric photovoltaic (FE-PV) devices.

The ferroelectric and photovoltaic properties are the two fundamental properties of the BFO for FE-PV devices. Researchers are investigating the specific factors that influence these properties. BFO is a multiferroic material that exhibits both ferroelectric and ferromagnetic behavior at room temperature, a large remanent polarization (∼100 μC/cm^2^),^4^ and a relatively narrow bandgap (∼2.5 eV), making it a unique choice for the absorber layer of the FE-PV solar cells. Further, BFO has potential applications in microelectronic memory and piezoelectric devices^5^ where the electromechanical and ferroelectric properties are controlled by the applied epitaxial strain. The magnetic properties of the BFO have potential application in magnetoelectric random access memory (MERAM), and its photocatalytic properties have application in solar water splitters.^6^ With the wide application of BFO, FE-PV devices have gained more attraction, as the coupling of two properties results in a high open-circuit voltage.

The polarization domain^7^ is one of the important parameters that influences the ferroelectric property of BFO, thereby affecting charge carrier separation. Another important parameter is the oxygen vacancy.^8^ Researchers^3,9^ were working on controlling the distribution of oxygen vacancies. The investigations were primarily aimed at the short-circuit photocurrent at different oxygen vacancies and hence the photovoltaic behavior of the BFO.^10–13^ It is evident from earlier works that BFO is a potential candidate for absorber layer application in perovskite solar cells.

Although BFO thin films have been widely investigated, prior studies have largely focused on UV detector applications^14,15^ and resistive-switching memory device^16,17^ applications rather than systematic compositional control of intrinsic defect chemistry. In particular, the influence of controlled Bi off-stoichiometry on the ferroelectric domain configuration and polarization-driven photovoltaic response remains insufficiently explored, especially in films grown by scalable solution-based techniques. In this work, we employ pulsed spray pyrolysis as a device-compatible and scalable approach to precisely tune the Bi content in BFO thin films and establish a direct correlation between compositional engineering, domain evolution, and ferroelectric photovoltaic performance. This integrated structure–domain–photoresponse analysis distinguishes this study from previous reports.

Furthermore, the device-quality film was studied under different Bi-to Fe-ratios, which in turn control the oxygen vacancy in the film and, as a result, the ferroelectric and photovoltaic outputs of the device. The Bi-to-Fe ratio was systematically varied; within the limit of the perovskite phase of the BFO, the bismuth content was varied from 5 atom % less to 10 at % excess. The effect of the Bi content on the mixed valence state of Fe and on oxygen vacancies was studied in detail to determine its impact on the ferroelectric and photovoltaic properties of the BFO film. The development of BFO films by a pulsed spray pyrolysis method and manipulating the oxygen vacancy through the Bi content play crucial roles in FE-PV devices. Hence, the study contributes to the advancement of scalable, device-quality fabrication of BFO thin films, which may potentially enhance energy harvesting techniques.

## Results and Discussion

Spray pyrolysis is a chemical technique that can be used for large-scale device fabrication. However, a rapid chemical reaction can lead to the formation of porous films. A schematic of the spray unit used for film deposition is shown in Figure 1a. Different deposition parameters, such as the flow rate, substrate-to-nozzle distance, air pressure, and substrate temperature, play crucial roles in determining film quality. Even after optimizing these parameters, the obtained bismuth ferrite thin film was porous, as evident from the field-emission scanning electron microscopy (FESEM) micrograph (Figure 1b). Then, the solution was sprayed onto the substrate in pulses, that is, a short spray time and then a small break to complete the thermal decomposition. The pulse deposition provides time for the particles to settle and complete the required thermodynamic reactions, resulting in a dense film. The film thickness was controlled by varying the number of pulses. This process resulted in the formation of larger grains and denser films, as observed in the FESEM micrographs (Figure 1c). The larger grain size in the pulsed spray-deposited films is attributed to the intentional pulse-off intervals, which allow for sufficient thermal relaxation, precursor decomposition, and enhanced surface diffusion. This reduces the nucleation density and promotes grain growth, thereby improving crystallinity. The X-ray diffraction (XRD) patterns of these films confirmed that pulsed deposition yielded well-crystallized, phase-pure BFO, matching the ICDD 01-080-3418 standard. An increase in the peak intensity by more than ten times and a decrease in the peak fwhm from 0.63(3)° to 0.56(5)° were clearly observed. The estimated crystallite size increased from approximately 14 to 27 nm, as determined using Scherrer’s formula, which substantiates the improvement in film quality achieved through pulsed deposition. An optimized pulsed deposition technique was employed in this study to achieve a more effective device using a cost-effective spray pyrolysis method.

**Figure 1:**
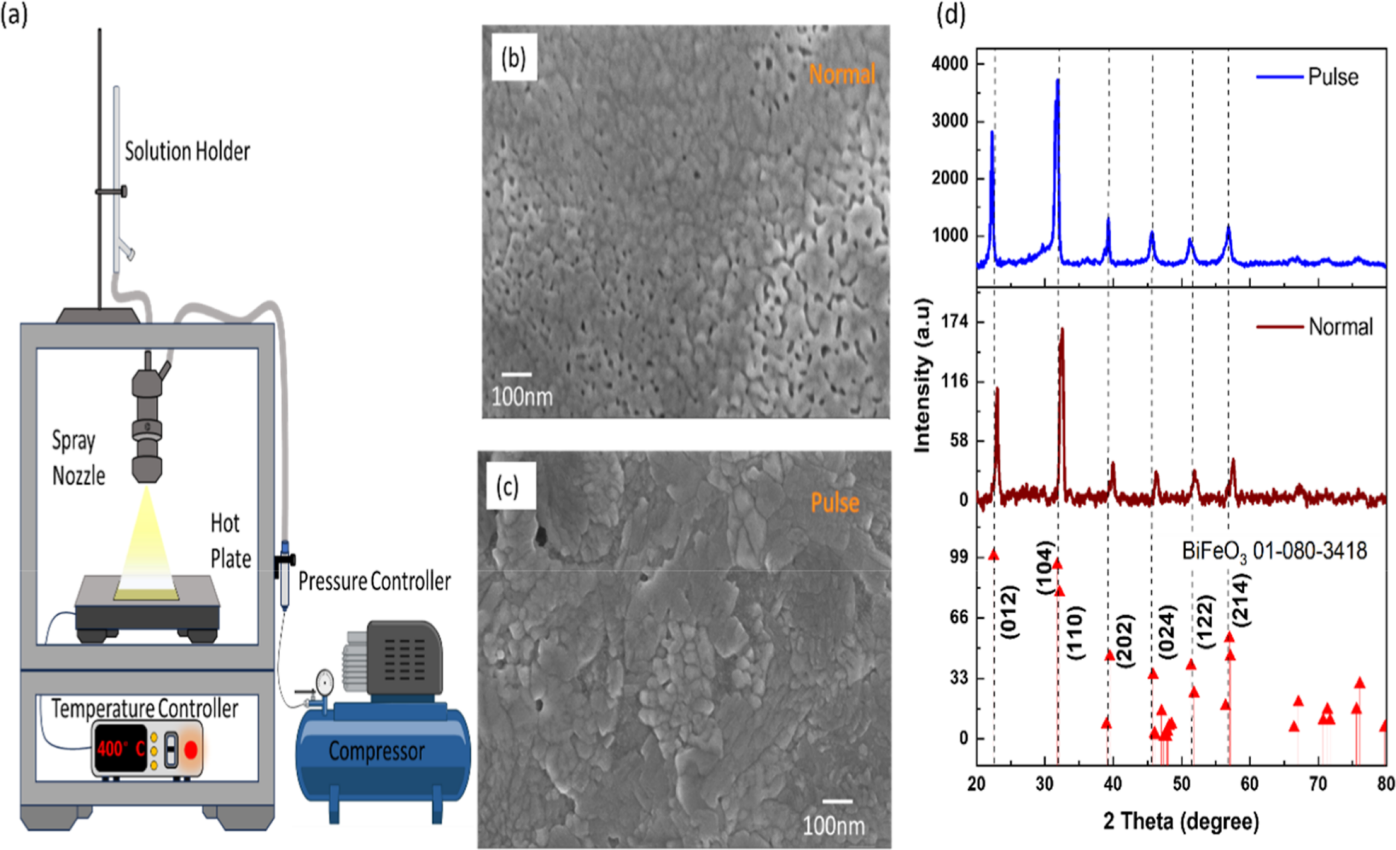
(a) The schematic diagram of the spray deposition unit; FESEM micrograph of the BFO film deposited by the (b) normal method and (c) pulsed spray method; (d) XRD pattern of the obtained BFO film.

The X-ray diffraction (XRD) pattern (Figure 2a) shows the samples with various nominal bismuth-to-iron ratios. The XRD patterns of all compositions can be indexed to the rhombohedral perovskite structure of BiFeO_3_ (ICDD 01-080-3418) without any detectable secondary reflections. No additional peaks corresponding to impurity phases, such as Bi_2_O_3_ or Fe_2_O_3_, were observed. Within the typical detection limit of laboratory XRD measurements, the films can therefore be considered single-phase. The substrate peaks are indicated by (*) to distinguish them from the film pattern. All films displayed consistent *R*3*c* symmetry with a rhombohedral crystal structure with lattice parameters of *a* = *b* = 5.58 Å, *c* = 13.85 Å, α = β = 90°, and γ = 120°. Notably, the crystal plane orientation changed with the bismuth content (Figure 2b). A lower bismuth content favored the clear splitting of the (1 1 0) and (1 0 4) planes, whereas a higher bismuth content resulted in the merging of the two peaks. In the Bi_1.05_FeO_3_ sample, the merging of two peaks into a single peak probably indicated reduced rhombohedral distortion, which is consistent with the findings of a previous study by Mocherla et al.^11^

**Figure 2:**
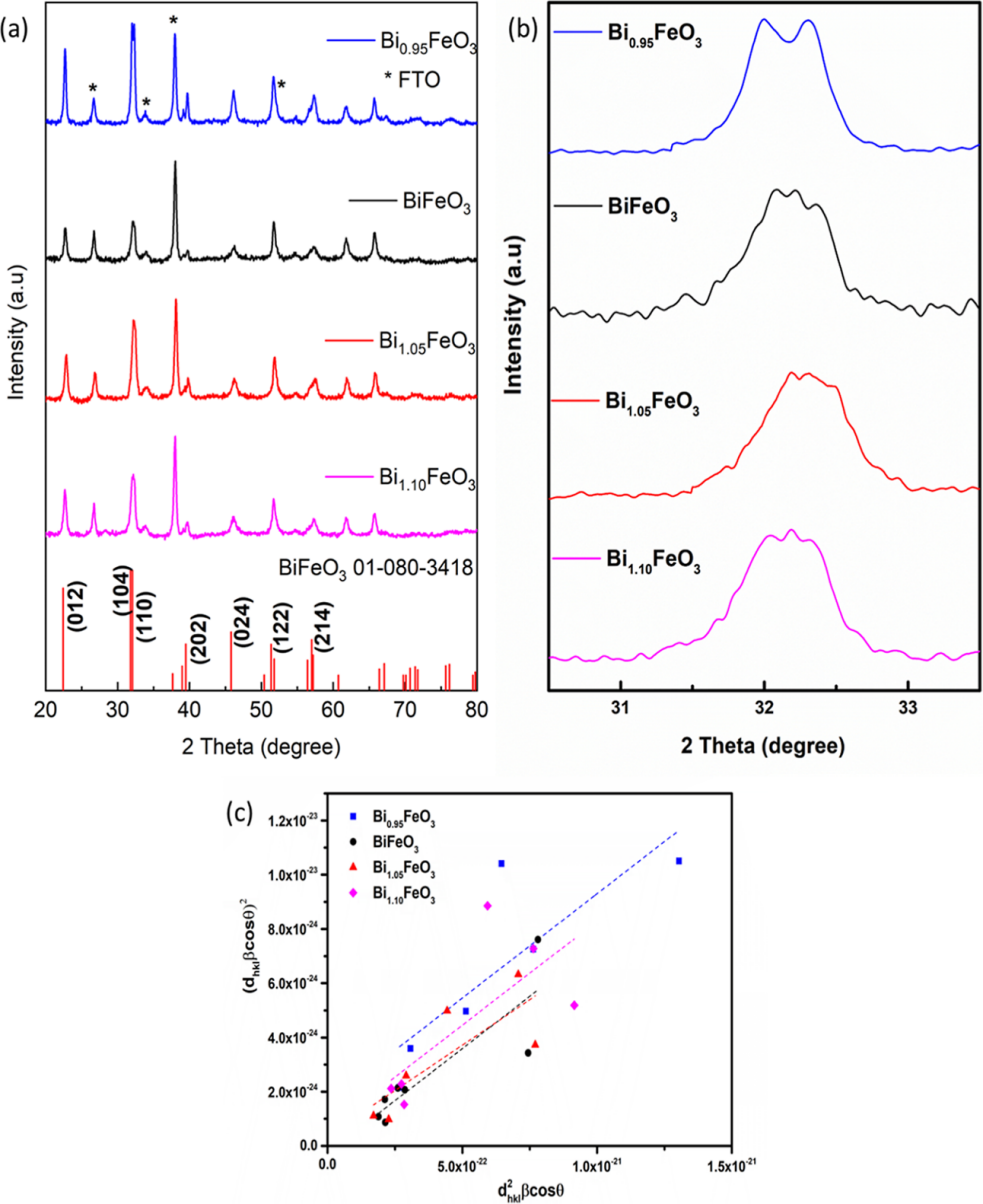
(a) X-ray diffraction (XRD) pattern of the BiFeO_3_ thin films prepared with different Bi concentrations, (b) enlarged portion of the XRD plot in the vicinity of 32°, and (c) size–strain plot for BiFeO_3_ samples with different Bi concentrations.

Peak fitting was performed by using a pseudo-Voigt function in OriginPro to accurately extract fwhm values. Then, the crystallite size was estimated using the Scherrer equation:

1 $$D=\frac{k\lambda }{\beta \;\cos\theta }\;\text{and}\;\Delta D=\frac{k\lambda }{\beta^{2}\;\cos\theta }\Delta \beta +\frac{k\lambda }{\beta {\;\cos}^{2}\;\theta }\sin\theta \cdot \Delta \theta$$

where *k* = 0.9 is the shape factor for the spherical or quasi-spherical crystallites, λ is the X-ray wavelength in Å, β is the fwhm, Δβ = 0.06° is the instrumental broadening, and θ is half the Bragg angle of the peak. The crystallite size of the most intense peak with error was calculated and is tabulated (Table 1). The crystallite size was verified using the size–strain plot (SSP) method as follows.

2 $${\left(d_{\left(hkl\right)}\beta_{\left(hkl\right)}\cos\theta \right)}^{2}=\frac{k\lambda }{D}\left(d_{\left(hkl\right)}^{2}\beta_{\left(hkl\right)}\cos\theta \right)+\frac{\epsilon^{2}}{4}$$

**Table 1:** Structural Parameters of BFO Samples from X-ray Diffraction Data

	crystallite size *D* (nm)		
sample	Scherrer’s method	SSP method	microstrain €
Bi_0.95_FeO_3_	14 ± 1	17	0.0089
BiFeO_3_	14 ± 1	17	0.0082
Bi_1.05_FeO_3_	15 ± 1	20	0.0081
Bi_1.10_FeO_3_	14 ± 1	18	0.0087

The crystallite size varied between 17 and 20 nm with variations in the Bi content (Table 1). The Bi_1.05_FeO_3_ sample had a crystallite size larger than that of the other samples. The excess bismuth substitution in the lattice initially increased the crystallite size and reached a maximum value for the Bi_1.05_FeO_3_ sample. Subsequently, the crystallite size decreased owing to off-stoichiometry, which is similar to the decrease in the crystallite size observed by Kumar and Kar.^13^ The observed variation in the crystallite size with an increasing Bi content can be attributed to the volatility and defect chemistry of bismuth ferrite. The earlier reports^12,18,19^ reported that high-temperature synthesis of BFO resulted in a bismuth-deficient phase due to the low thermal stability of the Bi. The addition of extra Bi compensated for these vacancies, promoting better crystallization and larger grain growth, as evidenced by the Bi_1.05_FeO_3_ sample. However, when the bismuth content was increased further (Bi_1.10_FeO_3_), surplus bismuth introduced lattice distortions and strain, which inhibited crystallite growth and reduced the grain size. This behavior is consistent with previous studies that reported a critical threshold of Bi content, beyond which the structural disorder dominated the defect compensation effects.^20^

A small induced strain in the structure can cause the tilting of interconnected octahedra, which may result in a drastic change in the physical properties owing to changes in the local crystal fields.^12^ Hence, the lattice strain was estimated by using the intercept of the size–strain plot (Table 1). The decrease in lattice strain is commensurate with the increase in crystallite size and suggests a reduction in imperfections in the crystal structure. Similar results were observed by Ahmad Wani et al.,^12^ who confirmed that the lattice strain decreased with increasing crystallite size.

The FESEM images (Figure 3a–d) of the deposited films clearly show uniform and continuous films without any holes or cracks. The dense black surface represents a thick and continuous film, and the enlargement of the grains with increasing bismuth content is clearly visible. The Bi_1.05_FeO_3_ sample exhibited a continuous and dense film compared to the other compositions. The addition of Bi reduced the number of grain boundaries in the film, resulting in a smoother film. Bismuth enhances the smoothness and continuity of the film and may improve charge transport.^21^ Furthermore, increasing the Bi content for Bi_1.10_FeO_3_ visibly reduced the grain size and resulted in the formation of more grain boundaries.

**Figure 3:**
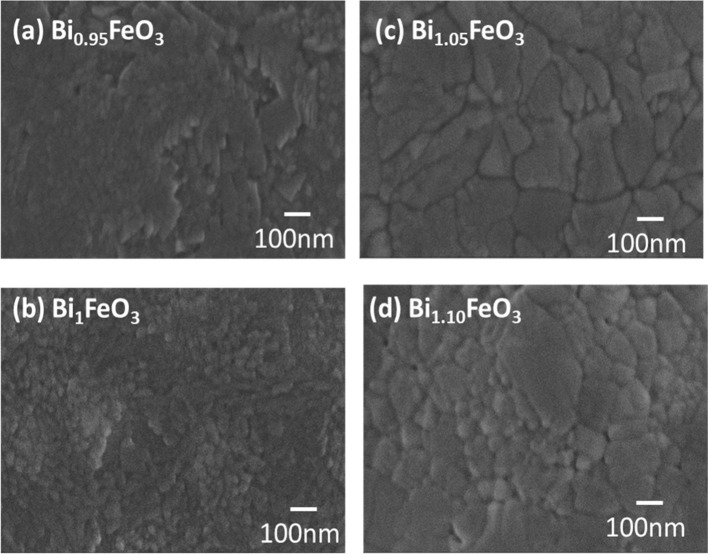
FESEM micrograph of bismuth ferrite films with different Bi concentrations.

The composition of the BFO film was analyzed using energy-dispersive spectroscopy (EDS), followed by X-ray photoelectron spectroscopy (XPS) for a more detailed analysis. The ratio of elemental composition (Bi/Fe) was estimated, which agrees with the expected ratios (Figure 4a). The elemental mapping (Figure S1) revealed a uniform distribution of elements in the film with no segregation observed near the boundaries.

**Figure 4:**
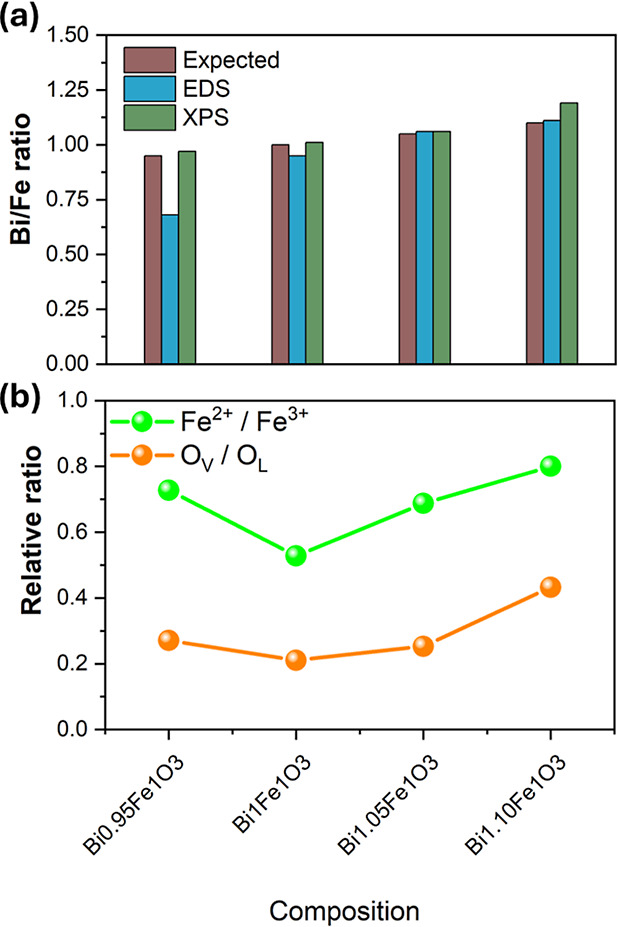
(a) Comparison of the Bi/Fe ratio for samples estimated using EDS and XPS. Similarly, (b) the relative fraction of Fe^2+^ with respect to Fe^3+^ and the oxygen vacancy peak with respect to the lattice oxide peak.

The oxidation state of the elements present in the film was studied by XPS. The XPS survey spectrum (Figure S2) confirms the absence of significant impurity peaks apart from adventitious carbon used for charge referencing. The presence of only Bi, Fe, and O in the film confirms its high purity. A detailed examination of the core spectra was conducted using CASA XPS software (version 2.3.26),^22^ and the results are presented in Figure 5 as well as in Tables S1–S4.

**Figure 5:**
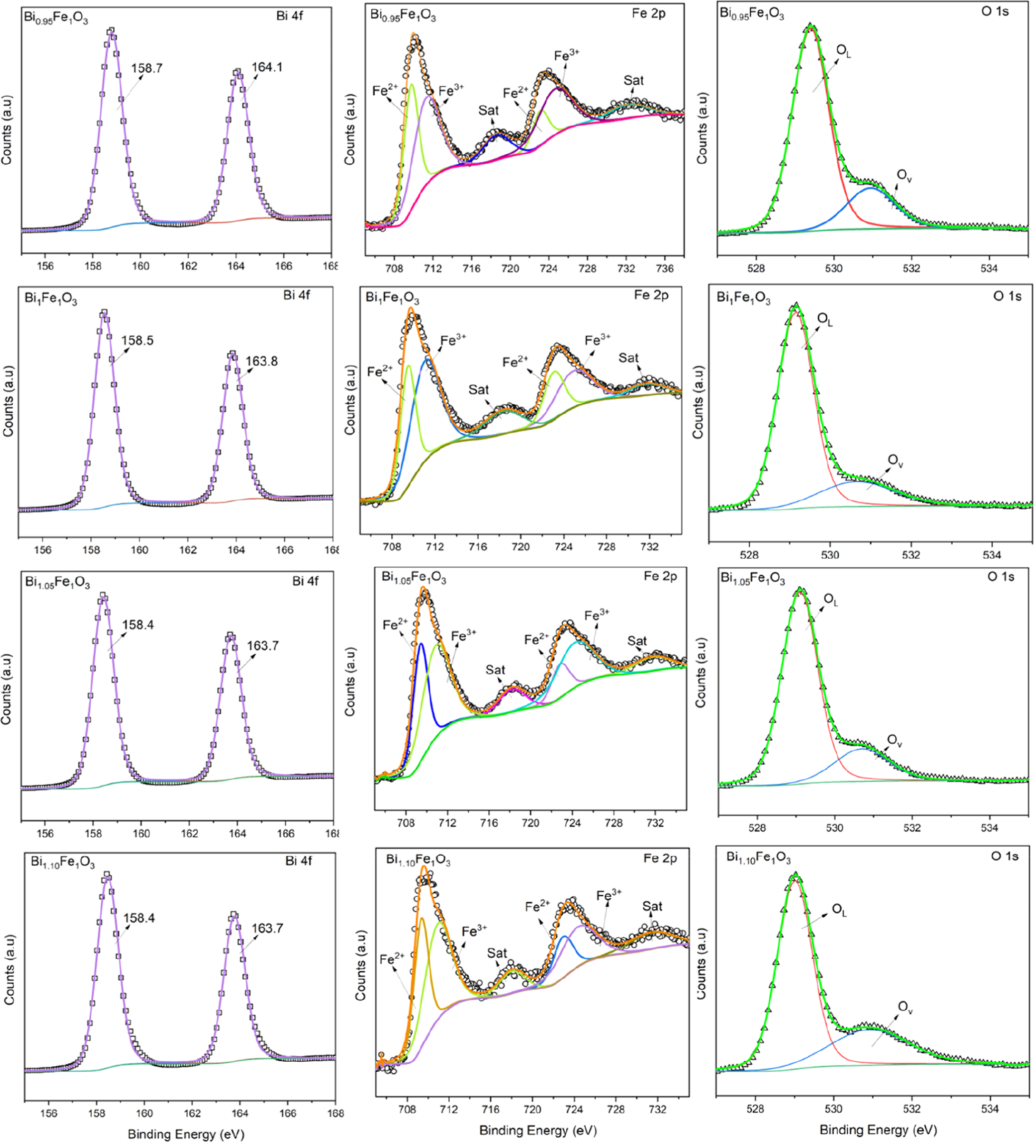
XPS core spectra of Bi 4f, Fe 2p, and O 1s for different BFO samples.

The Bi (Figure 5) is present in the +3 oxidation state with a doublet splitting of 5.31 eV between the Bi_7/2_ and Bi_5/2_, located at 158.7 and 163.8 eV, respectively. Hence, the study confirms the presence of only the Bi^3+^ state within the BFO film.^10^ The iron is present in two different oxidation states within the BFO lattice. The core spectra of Fe 2p (Figure 5) have two asymmetric peaks corresponding to spin–orbit splitting at 710.1 and 724.7 eV, with an energy difference of 14.6 eV. Similarly, the Fe 2p_1/2_ peak was deconvoluted into contributions from Fe^3+^ and Fe^2+^, and the details of the deconvolution are summarized in the table. The asymmetric peak can be resolved into two peaks corresponding to Fe^2+^ and Fe^3+^ states, with an additional satellite peak at 718.5 eV, confirming the mixed oxidation state of Fe.^23^ Furthermore, the area under the curve is quantified by the amount of each oxidation phase, and the ratio of Fe^2+^ to Fe^3+^ is illustrated in Figure 4b, which is directly related to the Bi content in the thin film.

The asymmetric oxygen O 1s spectrum was deconvoluted into multiple oxygen species. As indicated in Figure 5, a peak at 529.4 eV corresponds to the metal–oxygen bond (O_L_), and another at 532 eV is attributed to oxygen vacancies (O_V_).^24^ This dual-peak structure is characteristic of many metal oxide systems, providing valuable insight into defect chemistry. From the area under the peak, the maximum amount of oxygen vacancy is found to be 30% in the Bi_1.10_FeO_3_ sample. It is clear from Figure 4b that the off-stoichiometric sample has more oxygen vacancies than the stoichiometric sample; thus, the Bi content directly affects the oxygen vacancies in the film. At high temperature, the volatile Bi leads to the formation of both bismuth vacancy (*V*_Bi_^‴^) and oxygen vacancy (*V*_O_^‴^), which can be explained by Kröger–Vink notation^25^

$$2{Bi}_{Bi}^{x}+3O_{o}^{x}⇌2V_{Bi}^{\prime \prime \prime }+3V_{O}^{\prime \prime \prime }$$

To maintain charge neutrality, Fe^3+^ reduction occurs^26^

$$2{Fe}_{Fe}^{x}+V_{o}^{x}⇋2{Fe}_{Fe}^{\prime }+V_{O}^{\prime \prime }+\frac{1}{2}O_{2}$$

The elemental analysis clearly shows a variation in the mixed oxidation state of Fe and the amount of oxygen vacancies with changes in the Bi content in the film, as shown in Figure 4b. In the BFO lattice, Bi and Fe are anions, and O is the cation to maintain charge neutrality of the molecule. The increase in Bi atoms in the lattice reduces the oxidation state of Fe^3+^ to Fe^2+^ to balance the charge state of the molecule, while the creation of oxygen vacancies for charge compensation is another mechanism to maintain overall neutrality within the material.^27^ This balance between the iron oxidation state and the oxygen vacancy potentially contributes to the functional properties of the BFO film. The Bi_1_FeO_3_ sample has the least amount of oxygen vacancy and Fe^2+^ amount, while the off-stoichiometry results in an increase of both components, highlighting the complex charge-neutrality mechanism.^28,29^ Furthermore, by manipulating the Bi content, we can tailor the defect chemistry and hence alter the ferroelectric as well as the electric properties of the material according to the required application.

The electromechanical properties of the Bi_1_FeO_3_ and Bi_1.05_FeO_3_ thin films were studied by using piezoresponse force microscopy (PFM). PFM relies on the concept of the converse piezoelectric effect, wherein an applied electric field induces a strain in the ferroelectric (FE) material, allowing the FE domains to be visualized. The sinusoidal bias signal of frequency ω, applied to the tip, can be written as

3 $$E=E_{0}\;\cos\left(\omega t\right)$$

However, the strain produced by the FE samples is

4 $$d=d_{0}+D\;\cos\left(\omega t+\varphi \right)$$

Thus, we obtained the vertical and lateral PFM signals, along with their phases. The results (Figure 6) suggest that the deposited BFO films possess a complex ferroelectric domain microstructure.^30^ No significant correlations were observed among the topography, piezoresponse amplitude, or phase images. Consistent with the polycrystalline nature of the films, piezoresponses in both the in-plane (lateral PFM (LPFM)) and out-of-plane (vertical PFM (VPFM)) directions were observed. In the VPFM phase images, the color contrast (Figure 6) indicates the orientation of the out-of-plane polarization component. The domains with an out-of-plane polarization component normal to the surface and toward the top surface are depicted as yellowish, whereas the domains with downward polarization toward the bottom electrode are depicted as dark-brown. PFM measurements revealed the presence of randomly distributed polarization and domain walls across the grains of the film. Lattice defects usually accumulate at the domain walls, which can act as both conduction pathways and pinning centers for the domains.^31^ Domain walls typically act as conduction pathways for the photogenerated charge carriers.^32^ As shown in Figure 6, the Bi-rich sample has larger domains and fewer domain walls, resulting in fewer recombination pathways. Thus, the photocurrent was higher for the Bi_1.05_FeO_3_ sample than for the stoichiometric sample, as shown in Table 3. While the reduced domain wall density minimizes carrier trapping and recombination losses and improves the charge carrier transport, the dominant factor governing the enhanced photocurrent is the increased polarization coherence and strengthened internal ferroelectric field.

**Figure 6:**
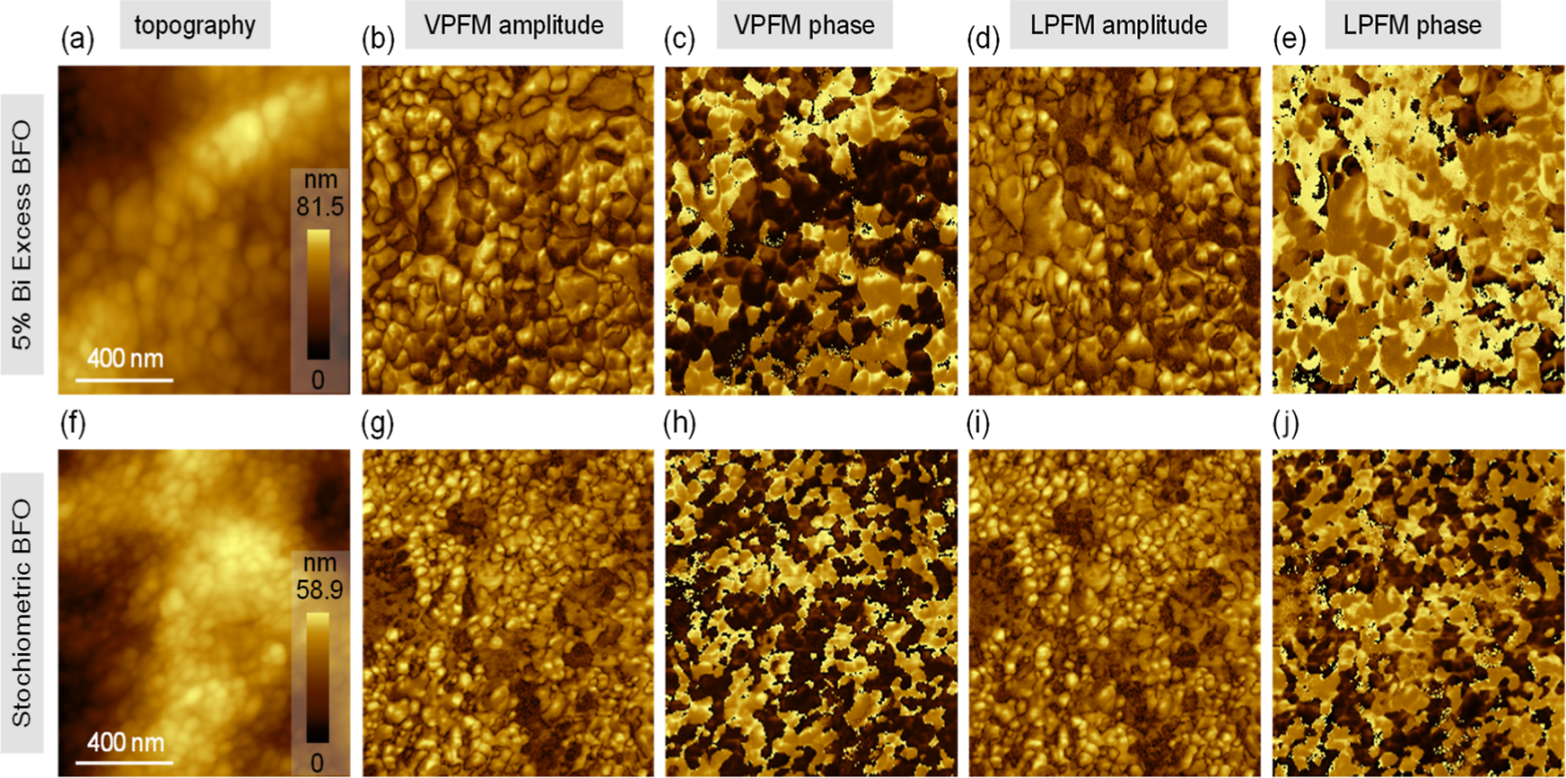
Piezoresponse force microscopy (PFM) mapping of Bi_1.05_FeO_3_ and Bi_1_FeO_3_ thin films: (a) topography and corresponding (b,c) vertical and (d,e) lateral PFM amplitudes and phases, respectively, for the Bi_1.05_FeO_3_ sample and (f) topography and corresponding (g,h) vertical and (i,j) lateral PFM amplitudes and phases, respectively, for the Bi_1_FeO_3_ sample.

PFM images confirmed the presence of randomly oriented ferroelectric domains in the bismuth ferrite thin films. Sharma et al.^33^ observed a similar ferroelectric domain distribution, and the random orientation of the grains in the films was the main reason for the random shapes and sizes of the polarization domains. Moreover, the Bi_1.05_FeO_3_ thin film appeared to have domains larger than those of the stoichiometric sample (Figures 6c and 6h).

Local piezoresponse spectroscopy (Figure 7) further revealed the switching of the domain orientation with the applied voltage in the Bi_1.05_FeO_3_ sample. The local vertical piezoresponse amplitude and phase loops exhibited electrically reversible bistable states along with sharp transitions at coercive voltages in the range of a few volts. The observed inhomogeneity in the domain microstructure and switching characteristics of the film likely affected the photogenerated charges and their response times.

**Figure 7:**
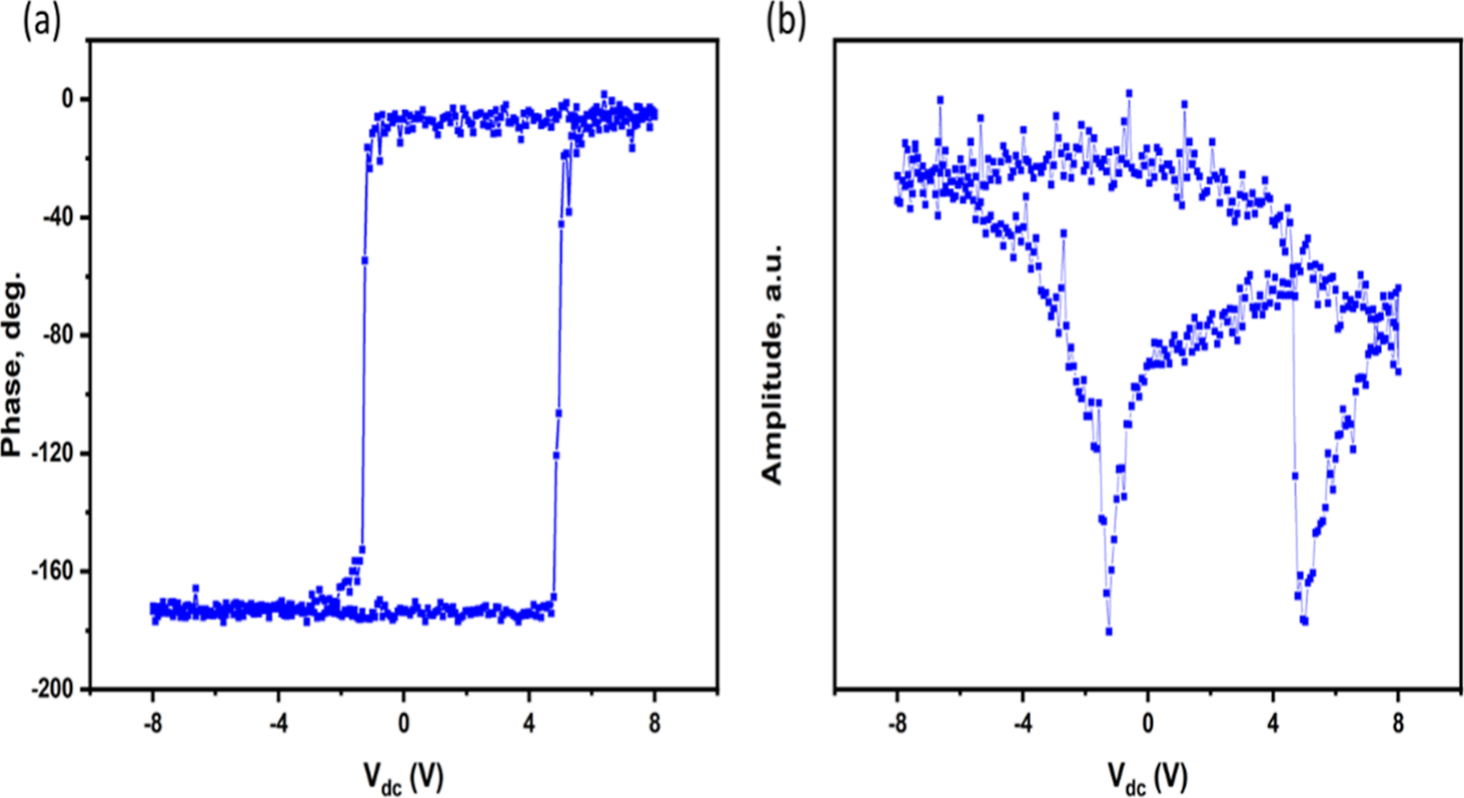
Bias-dependent local vertical PFM (a) phase and (b) amplitude spectroscopic measurements showing the polarization switchability of the Bi_1.05_FeO_3_ sample.

Theoretically, reducing the film thickness could increase the depolarization field and shorten the transit time of photogenerated carriers, thereby minimizing bulk recombination. However, this must be balanced against the potential for an increased leakage current and reduced optical path length. Future research can focus on a systematic thickness-dependent study to identify the critical thickness at which the carrier extraction efficiency is maximized without compromising the ferroelectric integrity of the BFO layer.

To quantify the local piezoelectric response, the switching spectroscopy PFM amplitude loops were converted to effective displacement using the product of amplitude and the cosine of the phase [amp × cos­(phase)]. The local piezoelectric coefficient (*d*_33_) was then obtained by normalization with respect to the AC drive voltage. For the Bi_1.05_FeO_3_ film, the effective remanent *d*_33_ is estimated to be ∼61 pm/V (Figure S3). The *d*_33_ is measured in OFF mode with minor electrostatic artifacts and shows true domain switching/hysteresis behavior. The measured *d*_33_ of ∼61 pm/V in the present work is consistent with previously reported local piezoelectric values for BFO-based ferroelectrics.^34–36^ This quantitative piezoelectric response further corroborates the intrinsic ferroelectric switching behavior observed in the PFM measurements.

The transmittance spectra (Figure 8a) of the BFO films show that the Bi_1_FeO_3_ film had the lowest transmittance in the visible region and that the transmittance increased with increasing bismuth content. Further, Bi_1.10_FeO_3_ samples show a decrease in transmittance. The absorption edge was blue-shifted with an increase in the Bi content. The increase in crystallite size reduces the perturbation in the band structure, thereby increasing the transmittance. The corresponding bandgap energy was calculated using Tauc’s relation.

5 $$\alpha h\nu =A{\left(h\nu -E_{g}\right)}^{n}$$

where *A* is a constant band-tailing parameter and *n* is an exponential factor that depends on the type of transition. Its value is 1/2 for a directly allowed transition and 2 for an indirectly allowed transition. Bismuth ferrite is a direct bandgap system;^37^ hence, using *n* = 1/2 in the above equation yields the energy bandgap of the material. The estimated energy band gap (Figure 8b and Table 2) shows a decrease in the energy band gap from 2.69 to 2.51 eV with an increase in bismuth content. The Bi_1.05_FeO_3_ sample had a large crystallite size and the smallest energy band gap of 2.51 eV, which is slightly lower than that reported previously.^38^ The energy band gap increases to 2.53 eV for the Bi_1.10_FeO_3_ sample, which may be due to the increase in the roughness of the film, as observed in the SEM images. Earlier results observed the same increase in bandgap with increasing roughness.

**Figure 8:**
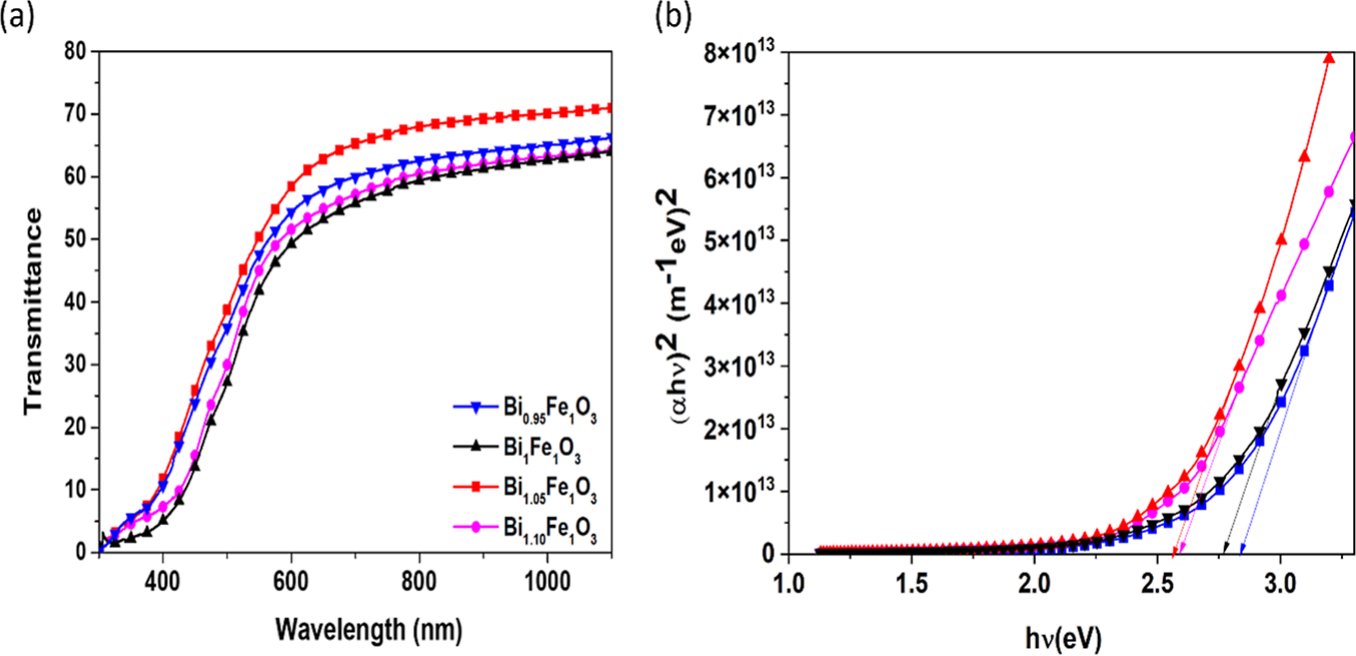
(a) Transmittance spectra and (b) Tauc’s plot for bismuth ferrite thin films with different Bi concentrations.

The slope of the logarithmic plot of the absorption coefficient with the incident energy provides the Urbach energy of the material. The Bi_1.05_FeO_3_ sample had the lowest Urbach energy (Table 2), and the deficiency of bismuth resulted in a high Urbach energy. The large crystallite size helps reduce the Urbach energy, because there are fewer defect states. However, the enhancement in the Urbach energy at higher Bi concentrations indicates that Bi affects the energy levels of this material.

**Table 2:** Optical and Electrical Parameters of the BiFeO_3_ Thin Films

sample	energy bandgap (eV)	Urbach energy (eV)	resistivity ρ (Ω cm) ×10^3^	carrier concentration *n* (×10^18^) (cm^–3^)	carrier mobility μ (cm^2^ V^–1^ s^–1^)
Bi_0.95_Fe_1_O_3_	2.69	0.837	4.7	3.7	6.3
Bi_1_Fe_1_O_3_	2.58	0.664	2.4	1.1	9.8
Bi_1.05_Fe_1_O_3_	2.51	0.659	1.6	4.8	10.5
Bi_1.10_Fe_1_O_3_	2.53	0.753	4.1	2.5	69.2

The electrical parameters were extracted using the Van der Pauw method and the Hall effect setup. The data in Table 2 suggest that all the films have a low resistivity on the order of 10^3^ Ω cm, compared with the other reported values of 10^8^ Ω cm.^39^ All samples had carrier concentrations on the order of 1 × 10^18^ cm^–3^. Among the samples, Bi_1.05_FeO_3_ had the lowest resistivity and the highest carrier concentration. The bismuth ferrite sample exhibited p-type semiconducting behavior, with an increase in carrier mobility as the Bi concentration increased.

Oxide semiconductors are intrinsically doped systems that exhibit p- or n-type conductivity in the absence of dopants. They were self-doped, owing to the defects. Depending on the site at which the defect occurs, it can be a metal or an oxide site. In this case, hole hopping from Fe^3+^ to Fe^2+^ resulted in conductivity. This occurs via oxygen, that is, Fe–O–Fe. The carrier concentration can be correlated to the Fe^2+^ and the oxygen vacancy, which directly contribute to the conductivity.^26,28,29^ Hence, from the XPS study, we can conclude that Fe^2+^ and the oxygen vacancy are the primary causes of the reported large carrier concentration and high conductivity of the film.

The electrode is crucial for extracting photogenerated charge carriers. The disparity in the work functions of the two electrodes generates a depolarized field that facilitates the drift of charge carriers. Among the two electrode configurations, the asymmetric configuration develops a built-in internal field that helps charge separation, whereas in the symmetric configuration, the built-in field is weaker. Two distinct positive electrodes, aluminum (Al) and gold (Au), were employed in device fabrication to achieve an asymmetric electrode configuration with an FTO. Such asymmetry can enhance carrier separation and improve charge extraction in ferroelectric photovoltaic devices.

The top electrode was deposited through a shadow mask, forming patterned contact pads. Illumination was performed from the top side, allowing light to directly reach the exposed BFO regions and ensuring effective photogeneration within the active layer. Photoresponse analysis was conducted using a Xe lamp and an electrometer, as depicted in Figure 9a, under zero-bias conditions. Figure 9b,c present the transient photocurrent of bismuth ferrite, which was measured as shown in the inset. The observed dark current in the film can be attributed to the presence of oxygen vacancies in the sample, which is a common cause of leakage currents.^10^

**Figure 9:**
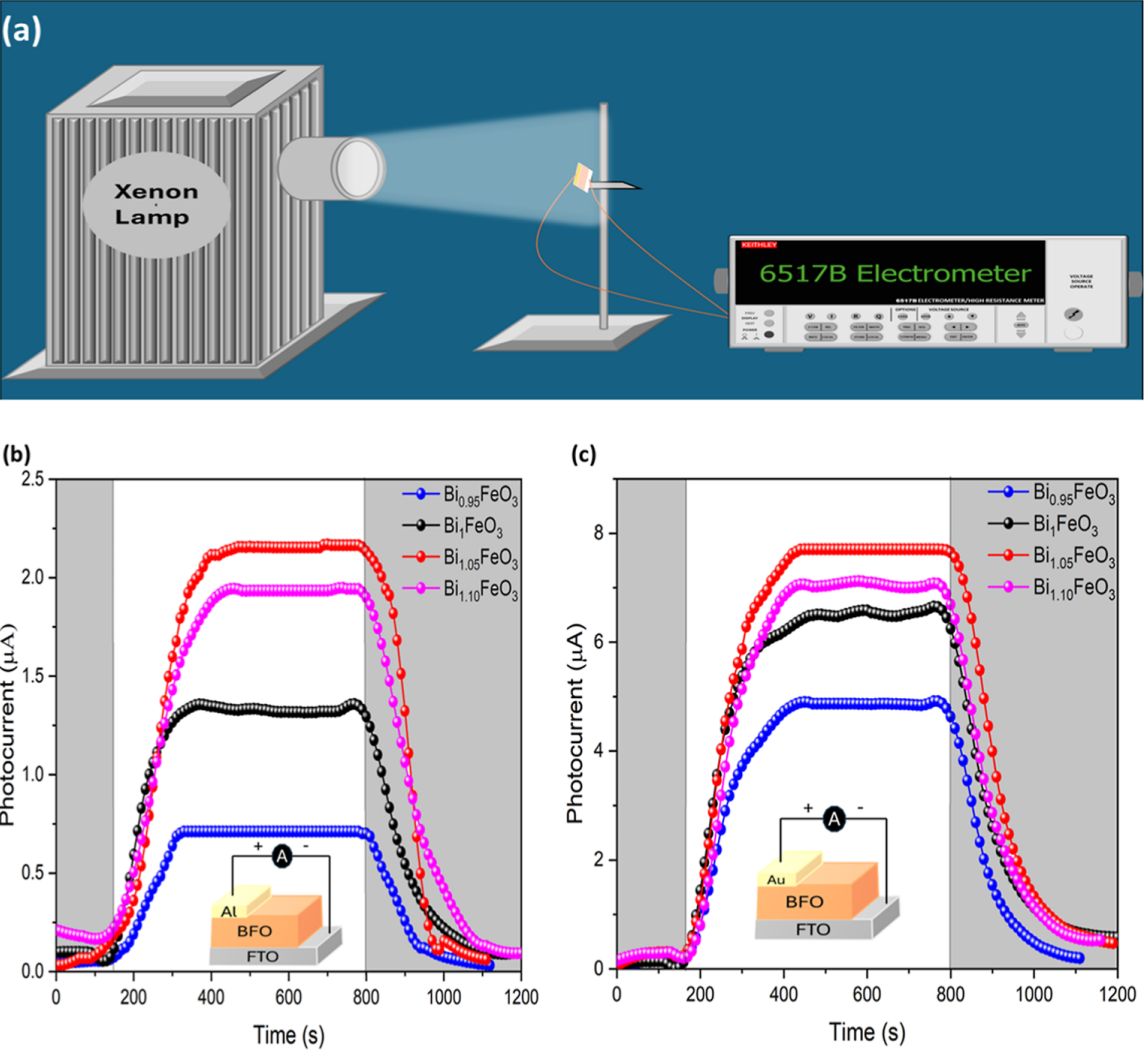
(a) Illustration of photoresponse measurement and (b,c) plot of photocurrent with respect to time for bismuth ferrite thin films with different Bi concentrations with Al and Au top electrodes, respectively.

With illumination of the light, the photocurrent gradually increases and reaches a maximum value. The longer response time and delay in establishing the steady-state photocurrent can be attributed to the diminishment of the imprint polarization field. The oxygen vacancy hinders the movement of charge carriers, and the initial energy is utilized to reduce the polarization field; as a result, a longer time is required for steady current attainment.^36^

Among the four samples, the Bi_1.05_FeO_3_ sample exhibits superior photoresponse properties. This can be attributed to the larger crystallite size, narrow energy band gap, and increased charge carrier concentration. Furthermore, the depolarization field induced by Fe^2+^ and oxygen vacancies facilitates charge carrier separation through the built-in electric field. All of these factors collectively resulted in a superior photoresponse from the Bi_1.05_FeO_3_ sample, regardless of the positive electrode considered.

The operational stability of the defect-rich oxide film was evaluated through prolonged illumination and repeated ON/OFF cycling tests (Figure 10a). The photocurrent remained stable for 32 min of continuous operation (Figure 10b) and showed negligible degradation across multiple illumination cycles, suggesting that oxygen vacancy migration and interfacial effects do not significantly impair device performance under the tested conditions.

**Figure 10:**
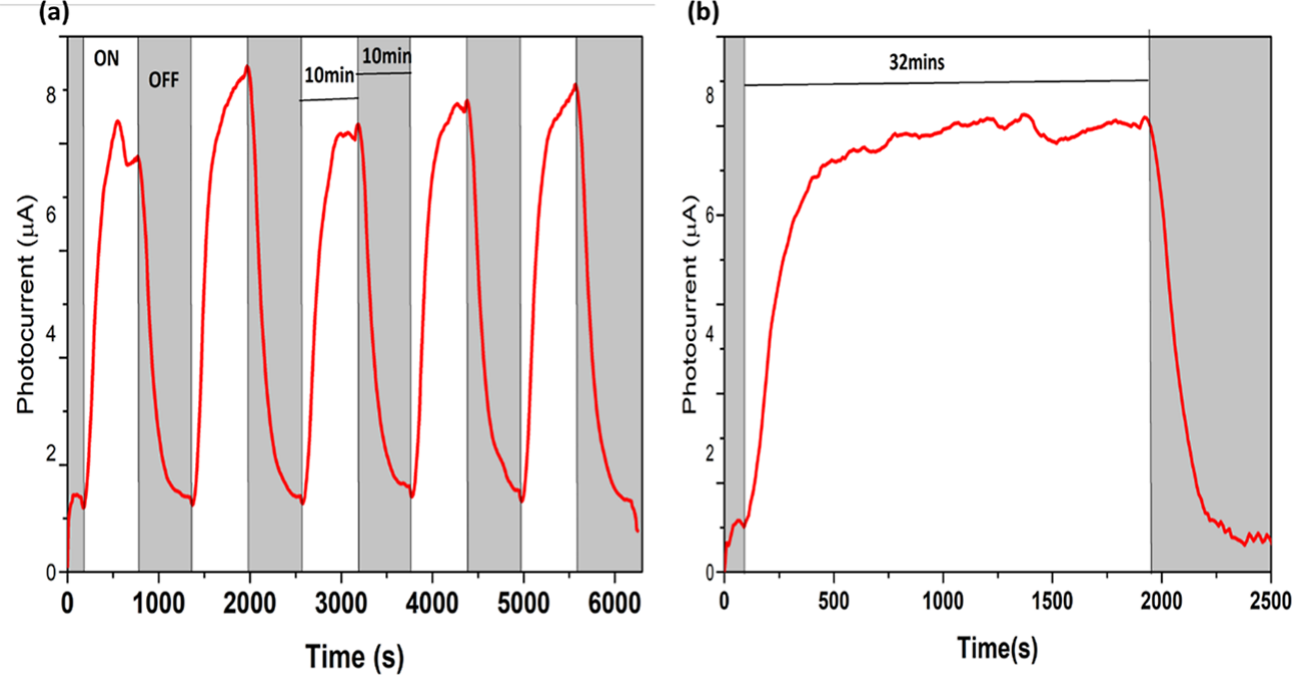
(a) Photocurrent measurement with multiple illumination cycles of light and (b) photocurrent for prolonged illumination for the FTO/Bi_1.05_FeO_3_/Au device.

Although the films exhibit measurable dark current associated with oxygen vacancies, several observations indicate that the dominant mechanism of photocurrent generation is the FEPV effect rather than defect-assisted leakage or photothermal contributions. First, the photocurrent is observed under zero external bias, demonstrating carrier separation driven by the internal polarization field. Second, the enhanced photoresponse correlates with improved ferroelectric domain coherence in the Bi_1.05_FeO_3_ film rather than with the highest dark current. Third, the photoresponse shows rapid and reproducible ON/OFF switching, inconsistent with slow thermal relaxation behavior. Furthermore, robust ferroelectric switching and *d*_33_ confirm the presence of a strong internal polarization field capable of driving directional charge separation. Together, these results support that polarization-induced photovoltaic effects dominate the observed photoresponse.

The photocurrent produced per unit watt of light illumination is called photoresponsivity. This crucial parameter in evaluating the photodetector performance was calculated using^40^

6 $$R=\frac{J_{sc}}{I}$$

where *J*_sc_ is the short-circuit current density and *I* is the intensity of the incident light (90 mW/cm^2^). This metric offers valuable insights into the device’s sensitivity and its ability to generate a measurable electrical response to incoming photons.

The calculated photoresponsivity of all of the samples, considering the Al electrode and the Au electrode, is tabulated in Table 3 along with the obtained short-circuit current density. The Bi_1.05_FeO_3_ sample exhibited the highest photoresponsivity of 0.173 mA/W with the Au electrode. Notably, this responsivity is higher than established ferroelectric materials such as lead–zirconate–titanate (PZT)^41^ and BaTiO_3_ ^42^ devices. Thus, the introduction of excess Bi favorably alters the electronic structure of the material to improve the responsivity of the material. Thus, the study highlights the contribution of the Bi content in the photoresponse property of the BFO film and paves the way for further investigations into the underlying mechanisms responsible for the observed enhancement.

**Table 3:** Optoelectronic Properties of BFO Samples

	short-circuit current density—*J*_sc_ (μA/cm^2^)		photoresponsivity—*R* (mA/W)	
sample	Al electrode	Au electrode	Al electrode	Au electrode
Bi_0.95_Fe_1_O_3_	1.8 ± 0.2	9.8 ± 0.3	0.021 ± 0.002	0.109 ± 0.003
Bi_1_Fe_1_O_3_	2.7 ± 0.3	13.1 ± 0.2	0.03 ± 0.003	0.145 ± 0.002
Bi_1.05_Fe_1_O_3_	4.3 ± 0.1	15.5 ± 0.2	0.048 ± 0.001	0.173 ± 0.002
Bi_1.10_Fe_1_O_3_	3.8 ± 0.2	14.4 ± 0.2	0.042 ± 0.002	0.159 ± 0.002

Table 3 shows that the device with a Au electrode yielded better results compared to the device with an Al electrode. This superior charge extraction can be attributed to the energy-band alignment between the BFO and the considered electrodes (Figure 11). The device with FTO and Al as the electrodes has a work function difference of 0.2 eV, whereas the device with FTO and Au electrodes has a work function difference of 0.9 eV. This larger work-function difference led to a larger depolarization field for charge-carrier separation. While the high work function of Au theoretically predicts a Schottky barrier, our experimental observations indicate a near-Ohmic contact. This transition is likely due to a high density of oxygen vacancies at the BFO surface. The presence of oxygen vacancies at the BFO surface can lead to Fermi-level pinning, modifying the Schottky barrier height. Despite this Ohmic behavior, the Au electrode remains superior because it maintains a higher internal field throughout the bulk of the BFO film than Al, thereby enhancing the collection efficiency of photogenerated carriers. Furthermore, the mismatch in the lattice or the presence of a thin interfacial layer at the FTO junction may introduce a non-negligible series resistance, contributing to the observed limitations in *V*_oc_.

**Figure 11:**
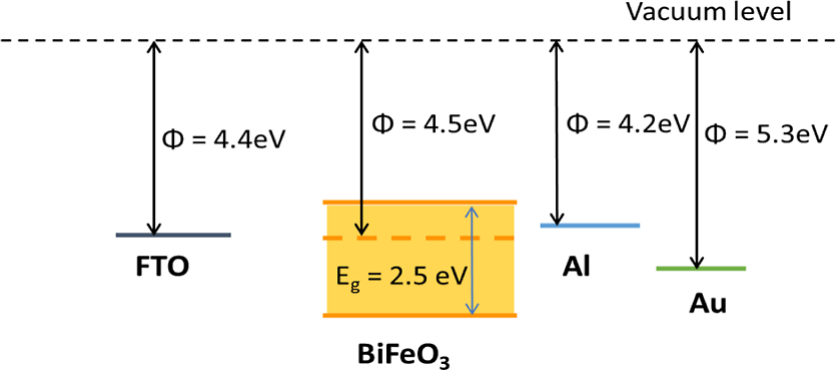
Energy level diagram of the BFO in comparison with the considered electrodes.

As shown in Table 4, the photovoltaic performance of BFO-based devices varies significantly, depending on the illumination source. For instance, the high *J*_sc_ values reported by B. Chen et al. (50 mA/cm^2^) and T. Yang et al. are attributed to the use of high-intensity monochromatic lasers (632 nm at 4500 mW/cm^2^ and 405 nm at 50 mW/cm^2^, respectively), which provide a high photon flux concentrated at wavelengths where the material may have specific absorption peaks. In contrast, our work utilizes a Xe lamp at 90 mW/cm^2^, which provides a broadband spectrum. This results in a lower, yet more representative, current density for solar energy harvesting applications.

**Table 4:** Comparison of Obtained Results with Published Results

device structure	short-circuit current—*J*_sc_ (mA/cm^2^)	light source and power	reference
ITO/PZT/ITO	50	632 nm	Chen et al.^43^
		4500 mW/cm^2^	
ITO/BFO/FTO	130	AM 1.5G	Dong et al.^44^
		100 mW/cm^2^	
Au/BFO/FTO	5.3 to 60	405 nm	Yang et al.^10^
		50 mW/cm^2^	
Au/BFO/Au	0.0016	532 nm	Yi et al.^45^
		20 mW/cm^2^	
Pt/BFO/FTO	0.012	purple laser	Zhou et al.^46^
		60 mW/cm^2^	
Au/BFO/FTO	0.015	Xe lamp^a^	this work
		90 mW/cm^2^	

a Multiple wavelength source.

It is found that the device gives an output comparable to that of devices fabricated by a physical deposition technique. Depositing device-quality film using a scalable, nonvacuum deposition technique presents numerous challenges. Proper optimization resulted in high-quality films using the pulsed spray pyrolysis technique. The output of the device is comparable to that of the devices fabricated by a vacuum-based technique. Thus, the study highlights the potential applications of the material and the deposition technique in commercial optoelectronics.

## Conclusions

A BFO thin film of high quality was deposited by using the pulsed spray pyrolysis technique. The modified method ensured a dense, crack-free film, as observed in the FESEM study. The high-quality film obtained by pulsed spray pyrolysis is further used to determine the effect of the Bi content on the properties of the BFO film. The effect of off-stoichiometry on the structural, optical, piezoelectric, and photoresponse properties of the film was discussed in detail. The off-stoichiometry introduced Fe^2+^ and oxygen vacancies in the film, which significantly alter both the functional and photoresponse properties. Notably, the sample with a 5 at. % excess of bismuth (1.05:1 Bi/Fe) exhibited enhanced conductivity and a higher carrier concentration compared to the other samples. Nanoscale piezoelectric studies confirmed the presence of larger, intricate ferroelectric domains in Bi_1.05_Fe_1_O_3_ than in the Bi_1_FeO_3_ samples. Enhanced device output with a Au top electrode, achieving a responsivity of 0.173 mA/W, is comparable to that of devices fabricated using vacuum-based deposition techniques. Thus, the study emphasizes the role of the Bi content in optimizing material properties and the role of the modified deposition technique in large-scale deposition.

## Materials and Methods

### Growth of the Device-Quality BFO Thin Film (Pulsed Spray Pyrolysis)

The synthesis of bismuth ferrite Bi_*x*_FeO_3_ (*x* = 0.95, 1, 1.05, and 1.10, corresponding to Bi_0.95_FeO_3_, BiFeO_3_, Bi_1.05_FeO_3_, and Bi_1.10_FeO_3_) films was carried out using bismuth­(III) nitrate pentahydrate [≥99%, Aldrich] and ferric nitrate nonahydrate [≥99%, Aldrich] in different ratios. Both chemicals were dissolved in double-distilled water to prepare a 0.01 M solution of each. This solution was stirred at room temperature and then sprayed onto cleaned F/SnO_2_ (FTO)-coated glass substrates by using the pulsed spray method. A constant distance of 28 cm is maintained between the substrate and nozzle. The substrate was maintained at 400 °C during deposition, followed by annealing at 450 °C for 5 h to achieve a film with better crystallization. The resulting films had thicknesses of 750 ± 50 nm, as measured using a stylus profilometer (Bruker DektakXT).

### Pulsed Deposition

High-quality BFO thin films were obtained on a substrate by pulsed spray pyrolysis. The films were deposited using a pulsed spray pyrolysis technique, with a spray duration of 1 min, followed by a 5 min interval between successive pulses (duty cycle ≈16.7%) to allow sufficient thermal recovery and solvent evaporation. The precursor solution was delivered at a flow rate of 1 mL min^–1^ by using a carrier gas pressure of 6 kg cm^–2^. The nozzle-to-substrate distance was maintained at 28 cm. A total of 20 mL of precursor solution was sprayed, corresponding to 20 pulses and a total deposition process time of approximately 2 h (including thermal recovery intervals). Under optimized conditions, films with an average thickness of ∼750 nm were obtained, with run-to-run thickness variation within ±50 nm.

### Characterization of the Thin Film

The structural properties were assessed using a Rigaku Miniflex 600 X-ray diffractometer with Cu Kα radiation (wavelength = 1.5418 Å). Continuous scanning from 20° to 80° at room temperature was performed at a step size of 0.01° for each of the samples. Field-emission scanning electron microscopy (FESEM) micrographs captured two-dimensional images of the film, focusing down to a width of 100 nm, with electron microscope settings of 5 kV EHT and 180k× magnification. The elemental composition was determined by using energy-dispersive X-ray spectroscopy (EDS). Furthermore, the elemental state was confirmed by X-ray photoelectron spectroscopy (XPS) in an ultrahigh-vacuum chamber equipped with a hemispherical analyzer utilizing Al/Mg X-ray sources. The spectra were recorded using an Al Kα line (1486.6 eV) at a takeoff angle of 90° and aligned using the C 1s peak (284.6 eV) as a reference. A Shirley-type background was employed for all of the regional scans. Peak modeling was performed with a consistent Gaussian–Lorentzian ratio to ensure reproducibility.^47^ The nanoscale piezoelectric properties, including polarization switching, were studied by utilizing piezoresponse force microscopy implemented on a commercial scanning probe system (AIST-NT SPM 1000). The response was acquired using an AC detection voltage of 1.0 V (peak-to-peak) with a frequency range of 400–1600 kHz, employing Pt-coated Si tips (*R* ∼ 30 nm, spring constant = 8 N/m) under a contact force of approximately 50–100 nN. Optical investigations encompassed transmittance and absorbance spectra acquired using a Shimadzu UV1800PC UV–vis–NIR double-beam spectrophotometer in the 300–1100 nm range at room temperature. The electrical properties were evaluated using the *Van der Pauw* method. Hall effect studies were conducted using a Keithley SMU 6220 instrument under a 0.6 T magnetic field. The measurements were performed with a 9.5 mA current, and the voltage was swept from −1 to 1 in steps of 0.02 V. Furthermore, the photoresponse of the device was studied using a Keithley electrometer unit (6517A) and a 90 mW/cm^2^ xenon lamp. A device with an illumination area of 0.5 cm^2^ was used for photoresponse measurements. Under zero-bias conditions, the photocurrent was measured as a function of the illumination time. Initially, the sample was kept in the dark for 2 min and then illuminated with light for 10 min. Furthermore, the sample was kept in the dark to obtain the *J*–*t* curve.

## Supplementary Material



## Data Availability

The data supporting this article are included in the Supporting Information.

## Notes

EDX spectrum and elemental mapping of Bi, Fe, and O for the Bi_1.05_FeO_3_ thin film; XPS survey spectra of the obtained samples; SS-PFM measurements in OFF mode for estimation of *d*_33_ in the Bi_1.05_FeO_3_ sample; and detailed XPS fitting parameters for Bi_0.95_FeO_3_, BiFeO_3_, Bi_1.05_FeO_3_, and Bi_1.10_FeO_3_ samples (PDF)
