# Supplementary material for: Pulsed Spray Pyrolysis for High-Quality Bismuth Ferrite Thin Films: Bi Content Enabled Tuning of Photoresponse, Ferroelectric Domains, and Charge Separation for Energy Harvesting Applications
Source: ACS Omega. 2026 Apr 14;11(16):24536–48. doi: 10.1021/acsomega.6c00654 (PMC13130109; doi:10.1021/acsomega.6c00654)
Supplement: Supplementary file 1 [file ao6c00654_si_001.pdf]

Supplementary Information: Pulsed Spray Pyrolysis for high-quality Bismuth Ferrite Thin Films: Bi content enabled tuning of photoresponse, ferroelectric domains and charge separation for energy harvesting applications

Nagashree Malur C[a], Haoze Zhang[b], Jan Seidel[c,d], Rajendra Bharathipura Venkataramana[a]*, Pankaj Sharma[b,c,e,] Vinayak B. Kamble[f], Suresh D. Kulkarni[g]*

[a] *Manipal Institute of Technology, Manipal Academy of Higher Education, Manipal -576104, India*

[b]*College of Science and Engineering, Flinders University, Bedford Park, SA, 5042, Australia*

[c]*ARC Centre of Excellence in Future Low-Energy Electronics Technologies, UNSW, Sydney, NSW, 2052, Australia*

[d]*School of Materials Science and Engineering, UNSW, Sydney, NSW, 2052, Australia*

[e]*Flinders Institute for Nanoscale Science and Technology, Flinders University, Adelaide, SA, 5042, Australia*

[f]*School of Physics, Indian Institute of Science Education and Research, Thiruvananthapuram, India*

[g]*Manipal Institute for Applied Physics, Manipal Academy of Higher Education, Manipal -576104, India*

[#]Corresponding authors: suresh.dk@manipal.edu (Suresh D Kulkarni), bv.rajendra@manipal.edu (Rajendra B V)

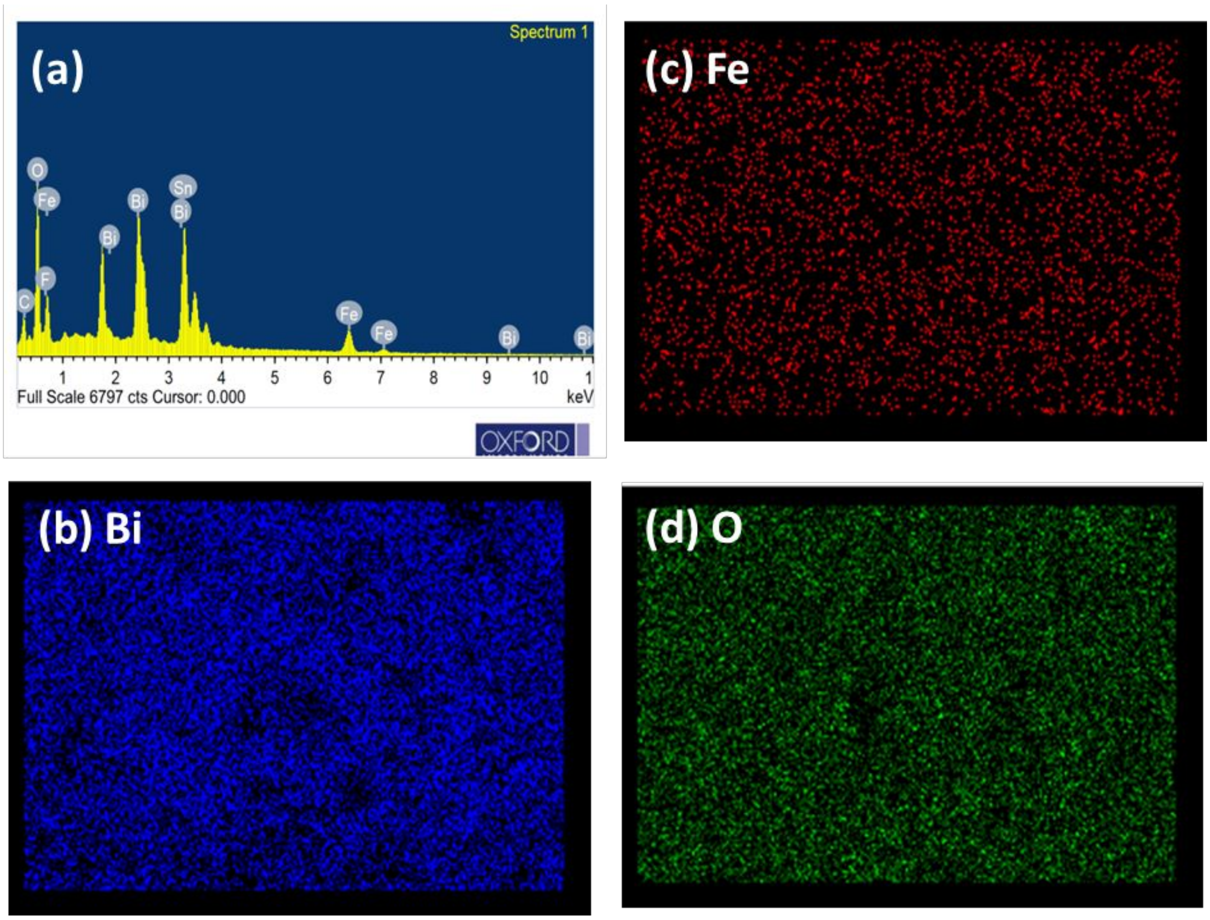

*Figure S*1: (*a) EDX spectrum and (b-d) elemental mapping of Bi, Fe and O for the* $Bi_{1.05}FeO_3$ *thin film*

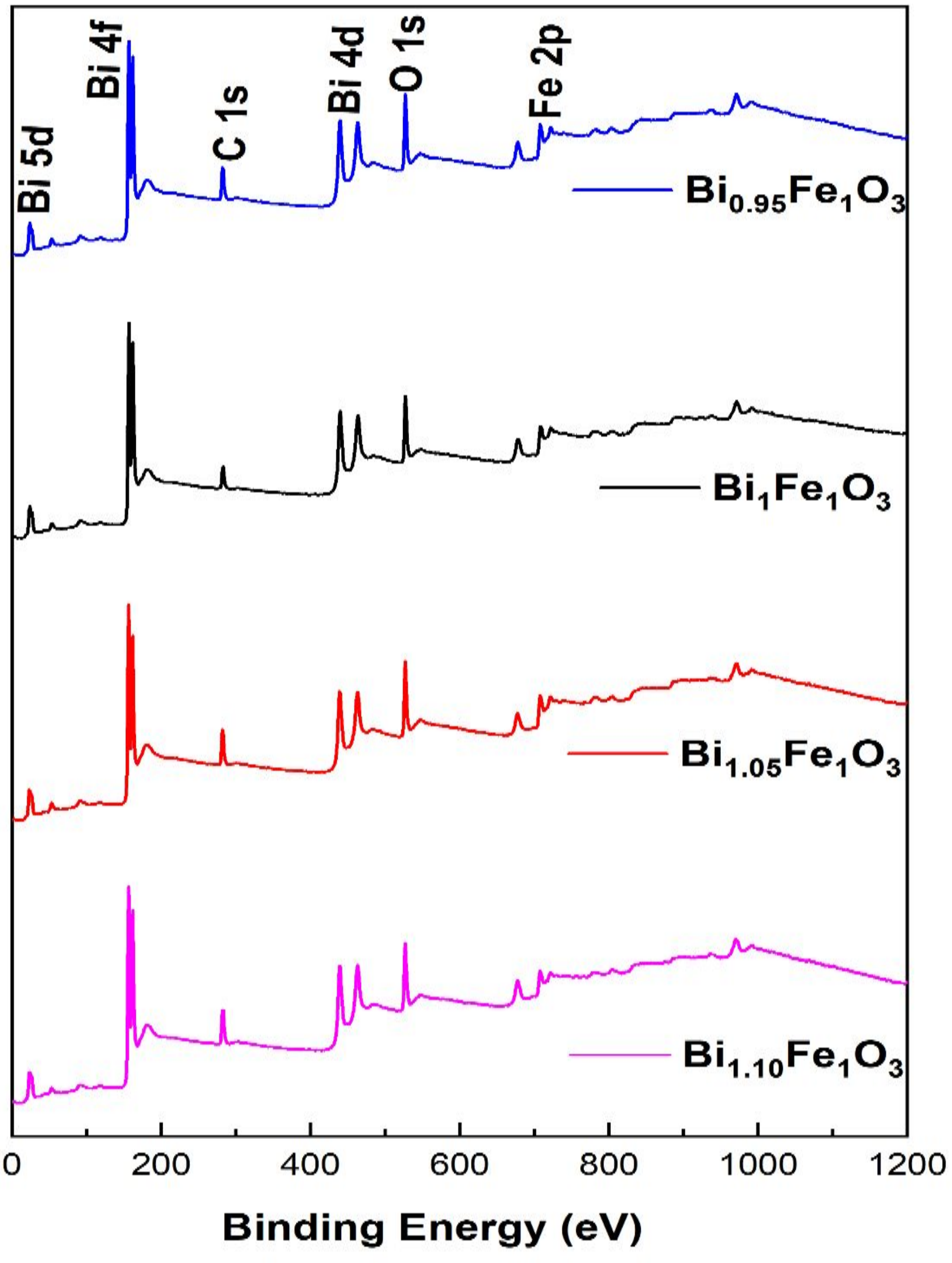

Figure S2: XPS Survey spectra of the obtained samples

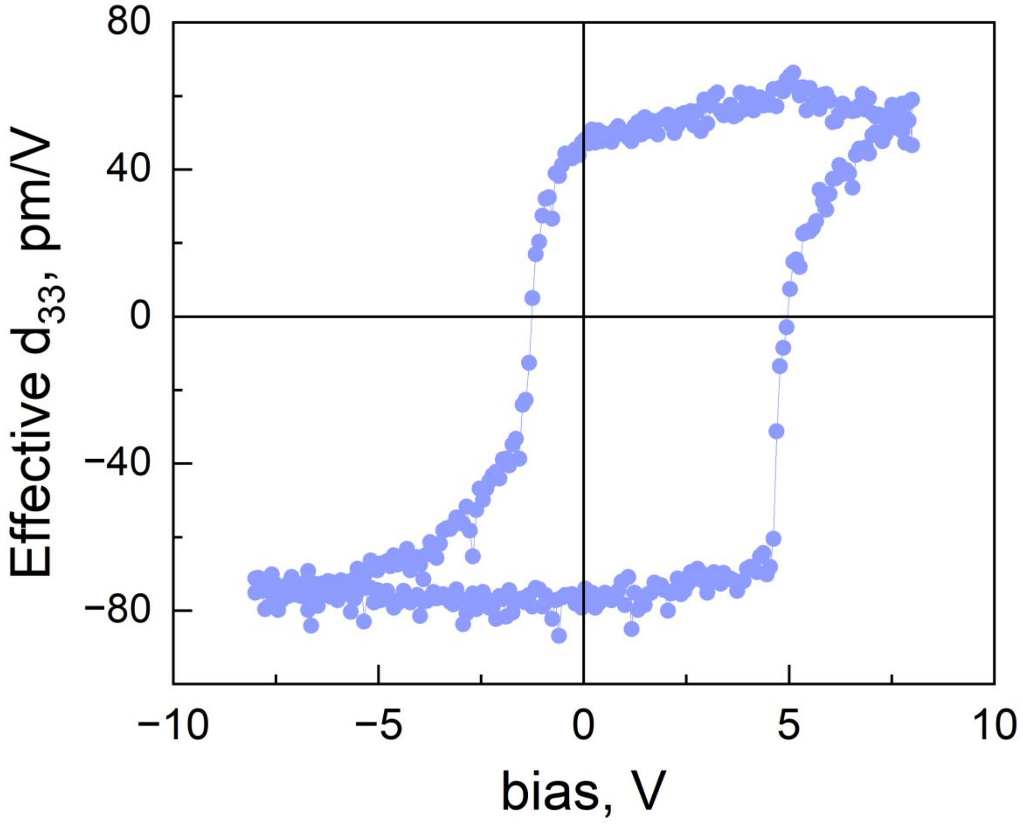

Figure S3: SS-PFM measurements in OFF mode to estimate d_{33} of the $Bi_{1.05}FeO_3$ sample

Table S1: Details of XPS fitting for the $Bi_{0.95}Fe_1O_3$ sample

Peak	Binding energy	FWHM	Area
Bi^{3+} - $4f_{7/2}$	158.7	1.07	56.97
Bi^{3+} - $4f_{5/2}$	164.1	1.07	43.03
Fe^{2+} - $2p_{3/2}$	709.4	1.66	21.86
Fe^{3+} - $2p_{3/2}$	711.1	3.24	35.34
Fe^{2+} sat - $2p_{3/2}$	718.3	4.19	10.46
Fe^{2+} - $2p_{1/2}$	723.0	2.25	11.74
Fe^{3+} - $2p_{1/2}$	724.9	4.03	16.06
Fe^{3+} sat - $2p_{1/2}$	731.7	3.62	4.55

O 1s - O_L	529.4	1.11	79.14
O 1s - O_V	530.95	1.46	20.86

Table S2:Details of XPS fitting for the $Bi_1Fe_1O_3$ sample

Peak	Binding energy	FWHM	Area
Bi^{3+} - $4f_{7/2}$	158.5	0.98	56.89
Bi^{3+} - $4f_{5/2}$	163.9	0.98	43.11
Fe^{2+} - $2p_{3/2}$	709.7	1.78	24.40
Fe^{3+} - $2p_{3/2}$	711.3	3.26	31.01
Fe^{2+} sat - $2p_{3/2}$	718.5	3.41	9.20
Fe^{2+} - $2p_{1/2}$	723.1	1.95	7.43
Fe^{3+} - $2p_{1/2}$	724.5	4.16	20.86
Fe^{3+} sat - $2p_{1/2}$	732.1	4.67	7.11
O 1s - O_L	529.1	1.04	79.43
O 1s - O_V	530.6	2.14	20.57

Table S3: Details of XPS fitting for the $Bi_{1.05}Fe_1O_3$ sample

Peak	Binding energy	FWHM	Area
Bi^{3+} - $4f_{7/2}$	158.4	1.04	57.13
Bi^{3+} - $4f_{5/2}$	163.7	1.03	42.87
Fe^{2+} - $2p_{3/2}$	709.4	1.67	22.43
Fe^{3+} - $2p_{3/2}$	710.9	3.18	33.30
Fe^{2+} sat - $2p_{3/2}$	718.2	3.06	7.98
Fe^{2+} - $2p_{1/2}$	722.8	2.04	7.86
Fe^{3+} - $2p_{1/2}$	724.2	4.29	21.89
Fe^{3+} sat - $2p_{1/2}$	731.7	4.12	6.54
O 1s - O_L	529.1	1.09	79.73
O 1s - O_V	530.7	1.63	20.27

Table S4: Details of XPS fitting for the $Bi_{1.10}Fe_1O_3$ sample

Peak	Binding energy	FWHM	Area

Bi^{3+} - $4f_{7/2}$	158.4	1.06	57.06
Bi^{3+} - $4f_{5/2}$	163.7	1.06	42.94
Fe^{2+} - $2p_{3/2}$	709.3	1.65	23.41
Fe^{3+} - $2p_{3/2}$	710.9	3.04	32.01
Fe^{2+} sat - $2p_{3/2}$	718.0	2.92	7.11
Fe^{2+} - $2p_{1/2}$	722.9	2.25	10.19
Fe^{3+} - $2p_{1/2}$	724.3	4.22	18.48
Fe^{3+} sat - $2p_{1/2}$	731.3	4.89	8.80
O 1s - O_L	529.0	1.05	70.40
O 1s - O_V	530.9	2.33	29.60
